# The impact of physical symptoms on depression and quality of life in patients with lung cancer: the moderating effects of illness perceptions and gender

**DOI:** 10.1186/s12955-025-02395-7

**Published:** 2025-06-23

**Authors:** Jinhuan Yang, Danni Dong, Gan He, Zhenghao Ge, Zhonglin Chen, Chenyan Han, Yi Zhao, Yaping He, Qiao Chu

**Affiliations:** 1 School of Public Health, Shanghai Jiao Tong University School of Medicine, No. 227 South Chongqing Rd, Shanghai, 200025 China; 2 Shanghai Chest Hospital, Shanghai Jiao Tong University, No. 241 West Huaihai Rd, Shanghai, 200030 China; 3 Center for Health Technology Assessment, Shanghai Jiao Tong University China Hospital Development Institute, Shanghai Jiao Tong University, No. 227 South Chongqing Rd, Shanghai, 200025 China

**Keywords:** Lung cancer, Physical symptoms, Depression, Quality of life, Illness perceptions

## Abstract

**Background:**

Physical symptoms negatively affect lung cancer patients’ emotional well-being and quality of life. It remains understudied about what psychosocial factors may buffer the negative impact of physical symptoms. This study examines how illness perceptions moderate the impact of physical symptoms on depression and quality of life, and further considers gender differences.

**Methods:**

A cross-sectional study was conducted on 316 lung cancer patients from Shanghai Chest Hospital in Shanghai, China, between July and September 2021. Participants completed questionnaires assessing physical symptoms, depression, quality of life, and illness perceptions.

**Results:**

Hierarchical regression analyses revealed significant two-way interactions between physical symptoms and illness timeline perceptions on both depression (β = 0.12, *P* = 0.028) and quality of life (β = -0.13, *P* = 0.010). Additionally, significant three-way interactions involving physical symptoms, illness perceptions, and gender were found. Specifically, illness consequences perceptions interacted with physical symptoms and gender to predict depression (β = 0.17, *P* = 0.012), while personal control (β = 0.14, *P* = 0.022) and treatment control (β = 0.17, *P* = 0.017) interacted similarly to predict quality of life. Simple slope analyses indicated that positive illness perceptions alleviated the negative effects of physical symptoms on depression and quality of life, particularly in females.

**Conclusions:**

These findings indicate that facilitating positive illness perceptions may buffer the adverse effects of physical symptoms on depression and quality of life. Personalized psychological interventions aimed at enhancing patients’ illness beliefs through positive psychological and behavioral strategies may contribute to improved coping and overall well-being.

## Background

According to the 2020 Global Cancer Report, the incidence and mortality rates of lung cancer rank first in China [1]. Lung cancer-related physical symptoms significantly impair patients’ emotional well-being and quality of life [2, 3]. Heavy symptom burden reduces patients’ treatment compliance, diminishes treatment outcomes, and leads to poor disease prognosis [4, 5]. Given these challenges, it is crucial to investigate potential psychosocial factors that may buffer the negative impact of physical symptoms on patients’ emotional well-being and quality of life. The present study aimed to investigate how patients’ illness perceptions moderate the association of physical symptoms with depression and quality of life, and further explore whether this moderating effect would further depend on patients’ gender.

Existing literature has documented an association between physical symptoms and depression, as well as quality of life, in lung cancer patients [6, 7]. The Common-Sense Model of Illness Self-Regulation [8–10] provides a theoretical framework for understanding this relationship, positing that illness perceptions as key mediators. Illness perceptions refer to individuals’ cognitive interpretations of the illness, shaped by their personal experiences, knowledge, values, beliefs, and needs [10]. According to the model, distressing cancer-related physical symptoms, such as pain and fatigue, can trigger negative illness perceptions, such as viewing the disease as uncontrollable or highly disruptive to their lives. These negative illness perceptions can then exacerbate emotional distress and further diminish the quality of life [8].

Although prior research has demonstrated the mediating role of illness perceptions in linking physical symptoms to psychological outcomes and quality of life [11–13], it remains unknown whether individual differences in illness perceptions also moderate this association. Individuals’ perceptions of illness are shaped by a variety of social and individual factors, such as personality, life experience, cultural beliefs, and social support [14]. Patients with more positive illness perceptions may interpret physical symptoms as controllable and less threatening fostering greater coping efficacy and confidence in treatment and recovery [15, 16]. This adaptive appraisal could mitigate the detrimental effects of symptoms on emotional well-being and quality of life. In contrast, patients with more negative illness perceptions may magnify the perceived threat of symptoms, interpreting them as unmanageable and life-disrupting. Consequently, these individuals are likely to experience heightened distress and poorer quality of life when faced with physical symptom burden [17].

Existing literature has demonstrated that a certain variable that acts as a mediator in a correlation may also serve as a moderator in the same relationship [18, 19]. For example, in a cross-sectional study on 249 undergraduate students, protective behavioral strategies mediated the relationship between self-regulation and drinking problems; at the same time, the authors also found that behavioral strategies moderated the relationship between self-regulation and drinking problems, with the use of protective behavioral strategies reducing the negative consequences of drinking for individuals with poor self-regulation [18]. Thus, individual differences in illness perceptions may also moderate the association between physical symptoms and depression, as well as quality of life.

### Current study

This cross-sectional study aimed to examine the moderating effect of illness perceptions on the association of physical symptoms with depression and quality of life, in lung cancer patients. We hypothesized that patients with more positive illness perceptions would experience fewer negative effects of physical symptoms on depression and quality of life compared to those with more negative illness perceptions. Furthermore, we explored whether the moderating effect of illness perceptions would further depend on patients’ gender.

Previous research has shown that when coping with cancer-related stress, female patients exhibit a greater tendency to seek social support from their social networks [20] and are more inclined to express their emotions compared to their male counterparts [21]. This active engagement in support-seeking and emotional expression may facilitate adaptive cognitive restructuring of illness——for example, by reframing the symptoms as manageable or transient through shared problem-solving or emotional validation. As a result, the buffering effect of illness perceptions is likely more pronounced among female cancer patients than male cancer patients.

## Methods

### Study design and participants

From July to September 2021, the study was carried out at Shanghai Chest Hospital in Shanghai, China. The hospital is well known for its treatment and research in lung cancer, so it attracts patients in need from all over the country. A convenience sampling technique was employed. Upon patients’ admission to the oncology department, the research team conducted a thorough review of the patient’s medical records to screen for eligibility. Patients who met the screening criteria were approached and informed about the study. Eligible and willing participants who provided their consent were enrolled and completed a cross-sectional survey measuring demographic information, clinical characteristics, and the variables of interest. This study was approved by the ethics committee at the School of Public Health at Shanghai Jiao Tong University School of Medicine (SJUPN-201915). All participants signed informed consent.

The inclusion criteria included: (a) diagnosed with lung cancer through cytological or histological examination; (b) age ≥ 18 years old; and (c) expected survival time of three months or more. The exclusion criteria included: (a) severe mental disorders or cognitive impairments; (b) poor language ability with inability to understand the study materials. A total of 350 patients were initially recruited for the survey. Ultimately, 316 patients agreed to participate and completed the questionnaire survey, resulting in a response rate of 90.3% (*n* = 350). The enrolled patients ranged from 24 to 81 years old (M = 61.87, SD = 9.57). Of this group, 74.7% (*n* = 316) were male, and 81.6% (*n* = 316) had an education level of high school or below. The average time since cancer diagnosis was 14.12 (SD = 22.16) months. Additional demographic and clinical characteristics are reported in Table 1.

**Table 1:** Demographic and clinical characteristics of 316 lung cancer patients

Variable	M (SD)	Frequency (%) ^a^
Age (years)	61.87(9.57)	
Gender		
Male	236	74.7
Female	80	25.3
Education		
Elementary school or lower	83	26.3
Junior high school	88	27.8
Senior high school	87	27.5
College or higher	58	18.4
Marital status		
Married	287	90.8
Single/divorced/widowed	29	9.2
Percentage of family income spent on cancer treatment for the past year		
< 50%	151	47.8
50–100%	45	14.2
> 100%	57	18.0
Occupation		
Full-time	22	7.0
Part-time	1	0.3
Sick leave/unemployed/retired	282	89.2
Months since diagnosis (month)	14.12(22.16)	
Cancer stage		
I	21	6.6
II	14	4.4
III	83	26.3
IV	163	51.6
Treatment received		
Chemotherapy	254	80.4
Surgery	106	33.5
Radiotherapy	53	16.8
Targeted therapy	117	37.0

^a^ Percentages may not add up to 100% because of missing data

### Measures

#### Physical symptoms

Physical symptoms were assessed via the Chinese version of the European Organization for Research and Treatment of Cancer Quality of Life Questionnaire for Lung Cancer Module (QOL-LC13) [22, 23], which comprises 13 items designed to assess 9 physical symptoms associated with lung cancer or its treatment, such as coughing, dyspnea, and chest pain. In this study, patients rated the severity of symptoms experienced in the past week on a scale ranging from (1) *not at all* to (4) *very much.* Because item 13 assesses pain relief with painkillers, the total score of the initial 12 items was used to assess the severity of physical symptoms, with higher scores indicating greater symptom severity. The internal reliability of the 12-item scale is 0.72 in this study.

#### Illness perceptions

Illness perceptions were evaluated using the Brief Illness Perception Questionnaire (BIPQ) [24, 25]. This questionnaire consists of 9 items, with each item representing an independent subscale designed to evaluate patients’ perception across various dimensions of their illness. In this study, the scoring for the four illness perception dimensions we utilized is as follows: (1) Illness consequences (How much does your illness affect your life? ) ranging from (0) *no affect at all* to (10) *severely affects my life*, with higher scores indicating more negative perceptions of illness consequences; (2) Illness timeline (How long do you think your illness will continue? ) ranging from (0) *a very short time* to (10) *forever*, with higher scores indicating more negative perceptions of illness timeline; (3) Personal control (How much do you feel you can control your illness? ) ranging from (0) *absolutely no control* to (10) *extreme amount of control*, with higher scores indicating more positive perceptions of personal control; and (4) Treatment control (How much do you think the treatment you are receiving, such as medication, can help control your illness? ) ranging from (0) *not at all* to (10) *extremely helpful*, with higher scores indicating more positive perceptions of treatment control.

#### Depression

Depression was assessed using the depression subscale of the Chinese version of the Hospital Anxiety and Depression Scale (HADS) [26, 27]. The depression subscale comprises 7 items, scored on a 4-point scale. Patients rated the severity of each symptom they experienced over the past week. Regarding the depression subscale, similar to our previous published study [28], the item-total correlation for item 10, “I have lost interest in my appearance” (*r* = 0.07), was significantly lower than the correlations for the other items in the subscale (ranging from 0.31 to 0.57). Furthermore, removing item 10 increased the internal reliability of the depression subscale from 0.65 to 0.74. Therefore, we decided to exclude item 10 and impute its score with the average score of the remaining 6 items. Based on the results of a previous validation study conducted in Chinese cancer patients [29], a total score of 8 was established as the cutoff point for probable diagnoses of depressive disorders.

#### Quality of life

The 5-level EQ-5D version (EQ-5D-5 L) was used to assess the quality of life [30]. The EQ-5D descriptive system comprises five dimensions: mobility, self-care, usual activities, pain/discomfort, and anxiety/depression, each with 5 levels ranging from (1) *no problems* to (5) *extreme problems*. Health utility values for the patients were calculated based on the Chinese residents’ health preference model [31]. Higher utility values represent better quality of life for patients. In the present study, the internal reliability of the scale is 0.68.

### Statistical analysis

Statistical analyses were performed using SPSS 26.0. Preliminary analyses included descriptive statistical analyses and correlation analyses among variables of major interest. Pearson correlations were performed to assess the associations between the variables of interest and sample characteristics to identify potential covariates to be controlled for in subsequent analyses.

To test the moderation of four dimensions of illness perceptions, we conducted four hierarchical regression models, one for each dimension of illness perceptions, separately. In step 1, potential covariates and the main effects of physical symptoms and illness perception were entered. In Step 2, the two-way interaction terms (physical symptoms × illness perception, physical symptoms × gender, illness perception × gender) were entered. In Step 3, we entered the three-way interactions among gender, illness perception, and physical symptoms. All variables were centered before the regression analyses to reduce multicollinearity. To decompose significant interactions, simple slope tests were conducted following the recommendations of Aiken and West [32] by comparing the associations of physical symptoms with depression and quality of life at high (M + 1SD) vs. low (M − 1SD) scores on illness perceptions, or between males and females.

## Results

### Preliminary analyses

Descriptive statistics and bivariate correlations of study variables were reported in Table 2. The average severity of physical symptoms was 166.72 (SD = 127.66). The average depression score was 3.11 (SD = 3.10). In this sample, 9.5% (*n* = 316) of patients were above the threshold for probable depressive disorder. As was shown in Table 2, there was a negative correlation between education level and depression (*r* = -0.13, *P* = 0.018), as well as a negative correlation between age and quality of life (*r* = -0.12, *P* = 0.046). Therefore, education level and age were included as covariates in subsequent hierarchical regression analyses.

**Table 2:** Descriptive statistics and correlations of study variables

Variable	Mean	SD	1	2	3	4	5	6	7	8	9
1. Physical symptoms	166.72	127.66	1.00	—	—	—	—	—	—	—	—
2. Illness consequences	5.78	3.13	0.26^**^	1.00	—	—	—	—	—	—	—
3. Illness timeline	5.71	3.23	0.21^**^	0.26^**^	1.00	—	—	—	—	—	—
4. Personal control	5.96	2.92	-0.20^**^	-0.20^**^	0.00	1.00	—	—	—	—	—
5. Treatment control	7.65	2.28	-0.24^**^	-0.10	-0.10	0.31^**^	1.00	—	—	—	—
6. Depression	3.11	3.10	0.33^**^	0.25^**^	0.21^**^	-0.22^**^	-0.22^**^	1.00	—	—	—
7. Quality of life	0.88	0.16	-0.49^**^	-0.26^**^	-0.19^**^	0.17^**^	0.18^**^	-0.34^**^	1.00	—	—
8. Age	61.87	9.57	0.13^*^	-0.06	0.10	-0.04	-0.07	0.08	-0.13^*^	1.00	—
9. Education	—	—	-0.08	0.05	-0.04	0.04	0.05	-0.12^*^	0.03	-0.30^**^	1.00

^*^P < 0.05, ^**^P < 0.01

### Regression analyses

#### Depression as the outcome variable

##### Two-way interaction between physical symptoms and illness timeline perceptions

**Fig. 1:**
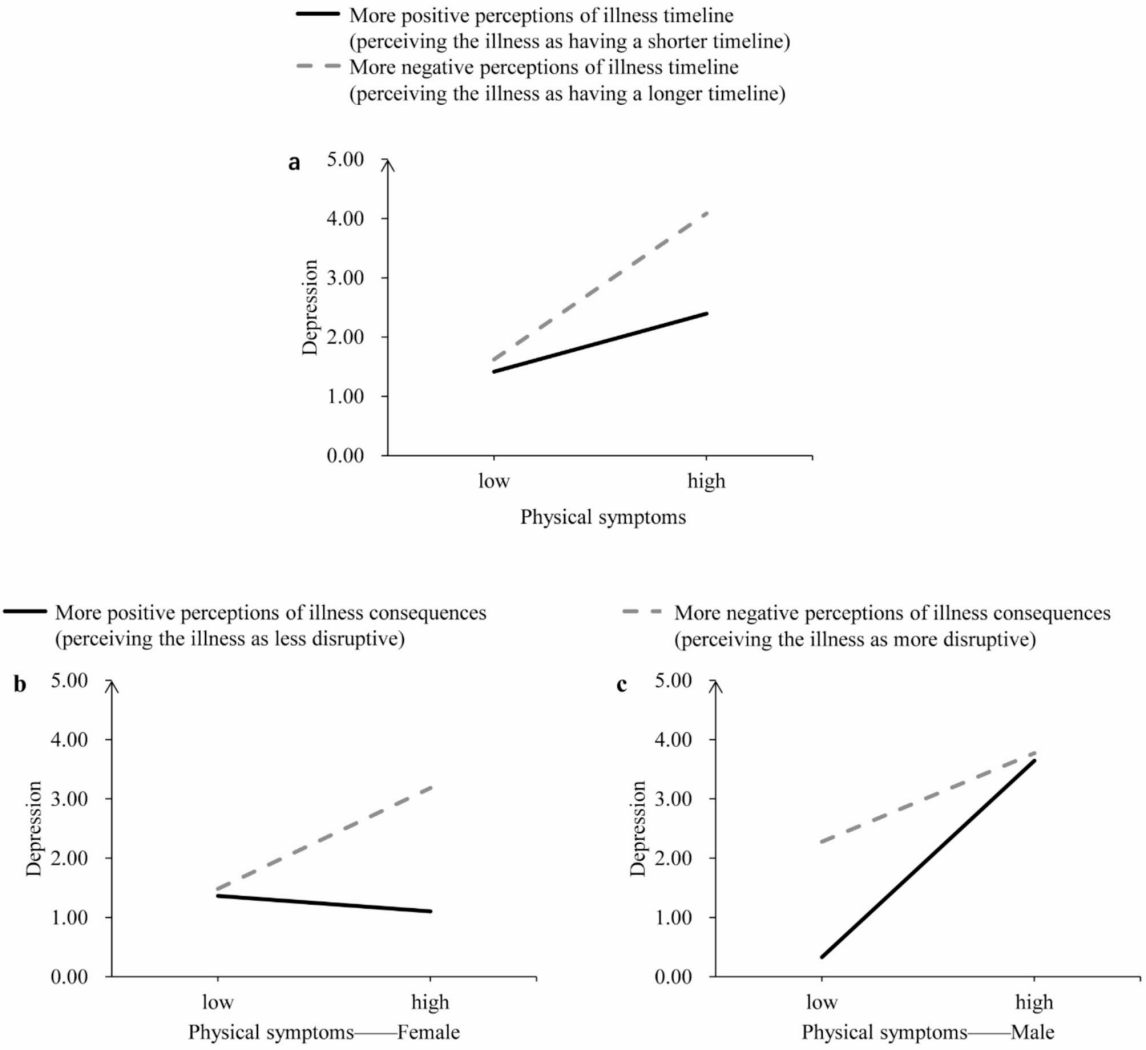
The two-way interaction effect of illness timeline perception and physical symptoms on depression (**a**); the three-way interaction effect of physical symptoms × illness consequences × gender on depression (**b**, **c**)

Table 3 presents the results of hierarchical regression analyses with four illness perceptions as moderators. There was a significant physical symptoms × illness timeline perceptions interaction (β = 0.12, *P* = 0.028). Simple slope test (see Fig. 1a) indicated that the association between physical symptoms and depression was stronger among patients with more negative perceptions of illness timeline (i.e., perceiving their illness as having a longer timeline) (β = 0.42, *P* < 0.001) compared to those with more positive perceptions (i.e., perceiving their illness as having a shorter timeline) (β = 0.18, *P* = 0.029). These results suggest that more positive perceptions of illness timeline may alleviate the detrimental effect of physical symptoms on depression.

**Table 3:** Hierarchical regression analyses of physical symptoms, illness perceptions, gender, and their interactions on depression

	β	*R* ^2^	ΔR^2^	df	F
**Illness consequences**					
Step 1		0.15	0.15	298	13.36^***^
Education	-0.11				
Physical symptoms	0.28^***^				
Illness consequences	0.18^**^				
Gender	-0.06				
Step 2		0.17	0.01	295	1.68
Physical symptoms × illness consequences	-0.04				
Physical symptoms × gender	-0.13				
Illness consequences × gender	-0.02				
Step 3		0.18	0.020	294	6.46^*^
Physical symptoms × illness consequences × gender	0.17^*^				
**Illness timeline**					
Step 1		0.14	0.14	276	11.41^***^
Education	-0.090				
Physical symptoms	0.30^***^				
Illness timeline	0.14^*^				
Gender	-0.05				
Step 2		0.17	0.03	273	3.20^*^
Physical symptoms × illness timeline	0.12^*^				
Physical symptoms × gender	-0.130				
Illness timeline × gender	-0.02				
Step 3		0.15	0.00	272	0.02
Physical symptoms × illness timeline × gender	-0.01				
**Personal control**					
Step 1		0.14	0.14	282	11.91^***^
Education	-0.09				
Physical symptoms	0.30^***^				
Personal control	-0.15^**^				
Gender	-0.05				
Step 2		0.16	0.01	279	1.21
Physical symptoms × personal control	0.03				
Physical symptoms × gender	-0.12				
Personal control× gender	0.03				
Step 3		0.16	0.00	278	0.32
Physical symptoms × personal control × gender	0.04				
**Treatment control**					
Step 1		0.14	0.14	290	11.83^***^
Education	-0.09				
Physical symptoms	0.29^***^				
Treatment control	-0.14^*^				
Gender	-0.05				
Step 2		0.16	0.02	287	1.76
Physical symptoms × treatment control	0.05				
Physical symptoms × gender	-0.12				
Treatment control × gender	0.06				
Step 3		0.17	0.01	286	3.01
Physical symptoms × treatment control × gender	-0.13				

^*^P < 0.05, ^**^P < 0.01, ^***^P < 0.001

##### Three-way interaction among physical symptoms, illness consequences perceptions, and gender

There was a significant physical symptoms × illness consequences perceptions × gender interaction (β = 0.17, *P* = 0.012). Simple slope analyses (see Fig. 1b and c) showed that among female patients, those with more positive perceptions of illness consequences (i.e., perceiving their illness as less disruptive) physical symptoms were not significantly associated with depression (β = -0.02, *P* = 0.910); conversely, for those with more negative perceptions of illness consequences (i.e., perceiving their disease as more disruptive), physical symptoms were positively associated with depression (β = 0.27, *P* = 0.042). In contrast, among male patients, regardless of their illness consequences perceptions, physical symptoms were positively associated with depression (all *P* < 0.05). These results suggested that positive perceptions of illness consequences may buffer the detrimental effect of physical symptoms on depression, but only among female patients.

#### Quality of life as the outcome variable

##### Two-way interaction between physical symptoms and illness timeline perceptions

**Fig. 2:**
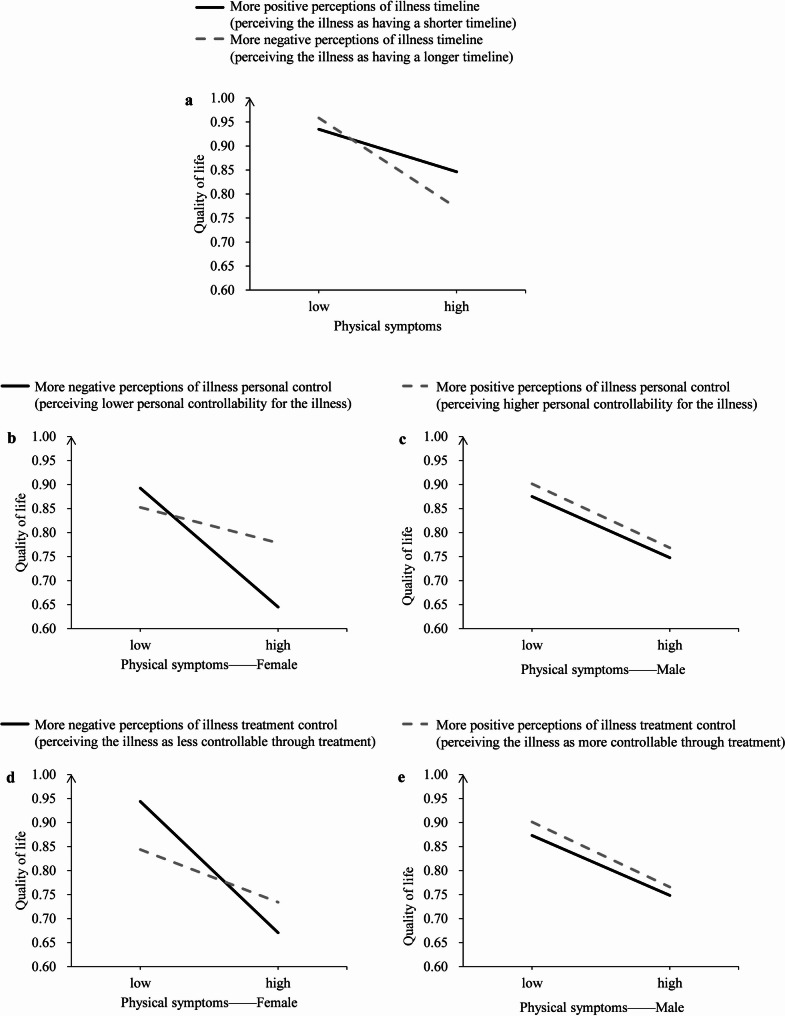
The two-way interaction effect of illness timeline perception and physical symptoms on quality of life (**a**); the three-way interaction effect of physical symptoms × personal control × gender on quality of life (**b**, **c**); the three-way interaction effect of physical symptoms × treatment control × gender on quality of life (**d**, **e**)

As is shown in Table 4, there was a significant physical symptoms × illness timeline perceptions interaction (β = -0.13, *P* = 0.010). As is shown in Fig. 2a, for patients with more negative perceptions of illness timeline (i.e., perceiving their illness as having a longer timeline), the association between physical symptoms and quality of life (β = -0.61, *P* < 0.001) was stronger than those with more positive perceptions of illness timeline (i.e., perceiving their illness as having a shorter timeline) (β = -0.34, *P* < 0.001). These results suggest that more positive perceptions of illness timeline buffer the detrimental effect of physical symptoms on quality of life.

**Table 4:** Hierarchical regression analyses of physical symptoms, illness perceptions, gender, and their interactions on quality of life

	β	*R* ^2^	ΔR^2^	df	F
**Illness consequences**					
Step 1		0.27	0.27	305	28.21^***^
Age	-0.09				
Physical symptoms	-0.43^***^				
Illness consequences	-0.15^**^				
Gender	-0.10				
Step 2		0.28	0.01	302	0.99
Physical symptoms × illness consequences	-0.06				
Physical symptoms × gender	-0.05				
Illness consequences × gender	-0.05				
Step 3		0.28	0.00	301	0.00
Physical symptoms × illness consequences × gender	-0.00				
**Illness timeline**					
Step 1		0.26	0.26	281	24.26^***^
Education	-0.07				
Physical symptoms	-0.46^***^				
Illness timeline	-0.09				
Gender	-0.10				
Step 2		0.28	0.02	278	2.51
Physical symptoms × illness timeline	-0.13^*^				
Physical symptoms × gender	-0.07				
Illness timeline × gender	0.01				
Step 3		0.28	0.00	277	0.93
Physical symptoms × illness timeline × gender	0.06				
**Personal control**					
Step 1		0.26	0.26	287	25.54^***^
Education	-0.08				
Physical symptoms	-0.46^***^				
Personal control	0.08				
Gender	-0.10				
Step 2		0.26	0.01	284	0.97
Physical symptoms × personal control	0.07				
Physical symptoms × gender	-0.07				
Personal control× gender	0.02				
Step 3		0.28	0.01	283	5.30^*^
Physical symptoms × personal control × gender	0.14^*^				
**Treatment control**					
Step 1		0.25	0.25	292	24.91^***^
Education	-0.08				
Physical symptoms	-0.46^***^				
Treatment control	0.07				
Gender	-0.10^*^				
Step 2		0.26	0.01	289	0.70
Physical symptoms × treatment control	0.05				
Physical symptoms × gender	-0.08				
Treatment control × gender	0.01				
Step 3		0.27	0.02	288	5.79^*^
Physical symptoms × treatment control × gender	0.17^*^				

^*^P < 0.05, ^**^P < 0.01, ^***^P < 0.001

##### Three-way interaction among physical symptoms, illness personal control perceptions, and gender

As is presented in Table 4, there was a significant physical symptoms × illness personal control perceptions × gender interaction (β = 0.14, *P* = 0.022). As is shown in Fig. 2b and c, among female patients, for those with more positive perceptions of illness personal control (i.e., perceiving higher personal controllability for their illness) (β = -0.20, *P* = 0.149), physical symptoms were not significantly associated with quality of life; whereas for those with more negative perceptions of illness personal control (i.e., perceiving lower personal controllability for their illness), physical symptoms were negatively associated with quality of life (β = -0.86, *P* < 0.001). In contrast, among male patients, physical symptoms were negatively associated with quality of life regardless of perceptions of illness personal control (all *P* < 0.05). These results suggest that positive perceptions of illness personal control may buffer the negative detrimental effects of physical symptoms on quality of life, but only among female patients.

##### Three-way interaction among physical symptoms, illness treatment control perceptions, and gender

There was a significant physical symptoms × illness treatment control perceptions × gender interaction (β = 0.17, *P* = 0.017). As is shown in Fig. 2d and e, among female patients, the association between physical symptoms and quality of life was stronger (β = -0.97, *P* < 0.001) for those with more negative perceptions of illness treatment control (i.e., perceiving the illness as less controllable through treatment) than those with more positive perceptions of illness treatment control (i.e., perceiving the illness as more controllable through treatment) (β = -0.33, *P* = 0.005). In contrast, among male patients, physical symptoms were negatively associated with quality of life regardless of perceptions of illness treatment control (all *P* < 0.05). These results suggest that the buffering effect of positive perceptions of illness treatment control was only significant among female patients.

## Discussion

The present study takes the first step in investigating the moderating role of illness perceptions on the association of physical symptoms with depression and quality of life in patients with lung cancer. We also examined the gender differences in this moderating effect. Our findings are generally in line with the hypotheses. We found that positive perceptions of the illness timeline buffered the negative impact of physical symptoms on depression and quality of life. Moreover, the buffering effects of the perception of illness consequences, personal control, and treatment control were significant only in female patients. Importantly, it should be noted that approximately 82.3% of the patients in our sample were in stages II to IV of lung cancer, indicating a predominance of advanced-stage cases. These patients often experience more severe physical symptoms and psychological burdens. In such contexts, palliative care plays a vital role—not only in managing symptoms and improving life expectancy but also in supporting emotional well-being and enhancing quality of life. In this context, our findings underscore the value of targeting patients’ illness perceptions as part of supportive interventions.

Our findings indicate that having more positive perceptions of the illness timeline (i.e., perceiving the illness timeline as short) helps alleviate the detrimental impact of physical symptoms on depression and quality of life. Patients with positive perceptions of their illness timeline tend to perceive the illness as temporary, controllable, and having a minimal impact on their lives, with effective treatment options. Recent research has uncovered a positive association between positive illness perceptions and milder depression, along with an improved quality of life [33]. These patients with positive perceptions tend to view the burden of symptoms as a relatively minor threat to their overall well-being [16]. This diminished perceived threat instills confidence in patients, enabling them to take effective measures in coping with their illness, thus mitigating the negative impact of physical symptoms on their emotional well-being and quality of life. In contrast, holding a negative attitude toward the illness may lead patients to be more sensitive to bodily discomfort, potentially escalating into a catastrophic perception of physical symptoms, thereby exacerbating depression and reducing the quality of life [34]. It is also plausible that individuals with a positive illness perception are more inclined to seek assistance from medical professionals when faced with symptoms [35]. Support from healthcare providers and other social support networks can enhance patients’ confidence in managing the illness, allowing for a more effective handling of physical symptoms.

Moreover, we found that the buffering effect of illness perception was only significant among female patients. One potential explanation may be related to gender differences in stress coping. Research has indicated that female cancer patients are more inclined to seek support from social networks [20]. Besides, female cancer patients show greater emotional expressivity than male patients [21]. Emotionally expressive coping has also been linked to improved psychological adjustment and physical health among breast cancer patients [36]. Through seeking support and expressing emotions with social partners, female patients likely have more opportunities to reappraise their disease from a more positive perspective. Consequently, the buffering effect of positive illness perceptions may be more pronounced among female patients. More studies are needed to confirm this finding.

### Implication

Our findings have implications for the development of interventions for managing symptoms and improving psychological adjustment of lung cancer patients. The results indicate that facilitating positive illness perceptions may be beneficial for reducing the negative impact of physical symptoms on depression and quality of life, especially for female patients. Thus, future interventions on lung cancer symptom management should incorporate a psychoeducation component on how to develop positive illness perceptions. Such psychoeducation can be delivered by trained oncology nurses, clinical psychologists, or social workers. In settings with limited access to mental health professionals, nurses, and other non-specialist staff can be trained to deliver brief, structured interventions, integrating them into routine care. Additionally, digital or remote psychoeducational programs may help increase accessibility and sustainability in real-world clinical practice. Moreover, given the finding that the buffering effect of illness perception is more salient among female patients, the design of the psychoeducation should also be tailored to the psychosocial needs of female patients. For example, nurses providing patients with health education about the disease and personalized emotional support can improve symptoms, self-management, and quality of life [37]. Mindfulness-based cognitive therapy aims to promote positive illness perceptions and beliefs that can reduce the level of negative illness perceptions, and improve psychological distress and quality of life [38].

### Limitations

The present study has several limitations. First, the study used a cross-sectional design, which resulted in correlational findings, making it impossible to conclude the association between physical symptoms, depression, and quality of life. Subsequent longitudinal studies are needed for further validation. Second, there is the reason for caution over the findings’ generalizability to other patient stages, given that the bulk of the study’s patients are in Stage III & IV. Further studies with a wider range of phases are necessary to confirm and extrapolate the results for different stages of the disease. Third, most participants had limited education (high school or lower), which may influence illness perception formation and limit generalizability. While our study found no significant associations of education level with illness perception dimensions, future studies should verify these findings in more educated populations, as cognitive aspects of illness representations may vary with educational attainment. Fourth, although the study was carried out in a renowned hospital that saw patients from all across the nation, the sample’s representativeness is limited because the study was conducted at a single center. Future multi-center studies that include a more diverse patient population may improve the external validity of the results. Finally, the use of EQ-5D-5 L—though chosen to minimize respondent burden and validated in Chinese populations—does not capture the multidimensional functional quality of life domains (e.g., role functioning and cognitive functioning). Future studies should integrate multidimensional quality of life measures, such as the European Organization for Research and Treatment of Cancer Quality of Life Questionnaire-Core 30 (EORTC QLQ-C30) to further examine how physical symptoms and illness perceptions interact with these specific quality of life dimensions.

## Conclusions

In conclusion, our study has demonstrated that facilitating positive illness perceptions can alleviate the negative impact of physical symptoms on depression and quality of life. Moreover, we found this moderating effect of illness perception was more salient in female patients. Future psychological interventions could target patients’ illness perceptions in a personalized manner, employing positive psychological and behavioral strategies to help patients better cope with the effects of the disease.

## Data Availability

No datasets were generated or analysed during the current study.

## References

[1] Sung H, Ferlay J, Siegel RL, Laversanne M, Soerjomataram I, Jemal A, Bray F. Global cancer statistics 2020: GLOBOCAN estimates of incidence and mortality worldwide for 36 cancers in 185 countries. CA Cancer J Clin. 2021;71(3):209–49. 10.3322/caac.21660 33538338

[2] Andersen BL, Valentine TR, Lo SB, Carbone DP, Presley CJ, Shields PG. Newly diagnosed patients with advanced non-small cell lung cancer: a clinical description of those with moderate to severe depressive symptoms. Lung Cancer. 2020;145:195–204. 31806360 10.1016/j.lungcan.2019.11.015 PMC7239743

[3] Mazor M, Paul SM, Chesney MA, Chen L-M, Smoot B, Topp K, et al. Perceived stress is associated with a higher symptom burden in cancer survivors. Cancer. 2019;125(24):4509–15. 31503333 10.1002/cncr.32477 PMC6891114

[4] Hu X, Walker MS, Stepanski E, Kaplan CM, Martin MY, Vidal GA, et al. Racial differences in patient-reported symptoms and adherence to adjuvant endocrine therapy among women with early-stage, hormone receptor-positive breast cancer. JAMA Netw Open. 2022;5(8):e2225485. 35947386 10.1001/jamanetworkopen.2022.25485 PMC9366541

[5] Pérol M, Winfree KB, Cuyun Carter G, Lin Cui Z, Bowman L, Garon EB. Association of baseline symptom burden with efficacy outcomes: exploratory analysis from the randomized phase III REVEL study in advanced non-small-cell lung cancer. Lung Cancer. 2019;131. 10.1016/j.lungcan.2019.03.001 31027699

[6] Shallwani SM, Simmonds MJ, Kasymjanova G, Spahija J. Quality of life, symptom status and physical performance in patients with advanced non-small cell lung cancer undergoing chemotherapy: an exploratory analysis of secondary data. Lung Cancer. 2016;99:69–75. 27565917 10.1016/j.lungcan.2016.06.018

[7] Poghosyan H, Sheldon LK, Leveille SG, Cooley ME. Health-related quality of life after surgical treatment in patients with non-small cell lung cancer: a systematic review. Lung Cancer. 2013;81(1):11–26. 23562675 10.1016/j.lungcan.2013.03.013

[8] Martin R, Rothrock N, Leventhal H, Leventhal E. Common sense models of illness: implications for symptom perception and health-related behaviors. In: Suls J, Wallston KA, editors. Social psychological foundations of health and illness. Blackwell series in health psychology and behavioral medicine. Malden: Blackwell Publishing; 2003. pp. 199–225.

[9] Leventhal H, Diefenbach M. Illness cognition: using common sense to understand treatment adherence and affect cognition interactions. Cogn Therapy Res. 1992;16:143–63.

[10] Leventhal H, Meyer D, Nerenz D. The common sense representation of illness danger. New York: Pergamon, pp. 17–30.

[11] Lee JY, Jang Y, Hyung W. Mediating effect of illness perception on psychological distress in patients with newly diagnosed gastric cancer: based on the common-sense model of self-regulation. Cancer Nurs. 2023;46(3):E138–45. 35324505 10.1097/NCC.0000000000001103 PMC10115492

[12] Miceli J, Geller D, Tsung A, Hecht CL, Wang Y, Pathak R, et al. Illness perceptions and perceived stress in patients with advanced gastrointestinal cancer. Psycho-oncology. 2019;28(7):1513–9. 31090125 10.1002/pon.5108 PMC6610754

[13] Chilcot J, Moss-Morris R. Changes in illness-related cognitions rather than distress mediate improvements in irritable bowel syndrome (IBS) symptoms and disability following a brief cognitive behavioural therapy intervention. Behav Res Ther. 2013;51(10):690–5. 23948131 10.1016/j.brat.2013.07.007

[14] Owadara FO. Cultural beliefs, social support and societal ascribed roles as determinants of patients’ perception of illness symptom. Int J Social Work. 2014;2:1–10.

[15] McGarry S, Girdler S, McDonald A, Valentine J, Lee S-L, Blair E, et al. Paediatric health-care professionals: relationships between psychological distress, resilience and coping skills. J Paediatr Child Health. 2013;49(9):725–32. 23808920 10.1111/jpc.12260

[16] Nie R, Han Y, Xu J, Huang Q, Mao J. Illness perception, risk perception and health promotion self-care behaviors among Chinese patient with type 2 diabetes: a cross-sectional survey. Appl Nurs Res. 2018;39:89–96. 29422183 10.1016/j.apnr.2017.11.010

[17] Valentine TR, Presley CJ, Carbone DP, Shields PG, Andersen BL. Illness perception profiles and psychological and physical symptoms in newly diagnosed advanced non-small cell lung cancer. Health Psychol. 2022;41(6):379–88. 35604701 10.1037/hea0001192 PMC9817475

[18] D’Lima GM, Pearson MR, Kelley ML. Protective behavioral strategies as a mediator and moderator of the relationship between self-regulation and alcohol-related consequences in first-year college students. Psychol Addict Behav. 2012;26(2):330–7. 22288975 10.1037/a0026942

[19] Karazsia BT, Berlin KS. Can a mediator moderate? Considering the role of time and change in the mediator-Moderator distinction. Behav Ther. 2018;49(1):12–20. 29405917 10.1016/j.beth.2017.10.001

[20] Goodwin BC, Chambers S, Aitken J, Ralph N, March S, Ireland M, et al. Cancer-related help-seeking in cancer survivors living in regional and remote Australia. Psycho-oncology. 2021;30(7):1068–76. 33534193 10.1002/pon.5643

[21] Zakowski SG, Harris C, Krueger N, Laubmeier KK, Garrett S, Flanigan R, et al. Social barriers to emotional expression and their relations to distress in male and female cancer patients. Br J Health Psychol. 2003;8(Pt 3):271–86. 14606973 10.1348/135910703322370851

[22] Bergman B, Aaronson NK, Ahmedzai S, Kaasa S, Sullivan M, EORTC Study Group on Quality of Life. The EORTC QLQ-LC13: a modular supplement to the EORTC core quality of life questionnaire (QLQ-C30) for use in lung cancer clinical trials. Eur J cancer (Oxford England: 1990). 1994;30a(5):635–42. 10.1016/0959-8049(94)90535-5 8080679

[23] Chie WC, Yang CH, Hsu C, Yang PC. Quality of life of lung cancer patients: validation of the Taiwan Chinese version of the EORTC QLQ-C30 and QLQ-LC13. Qual Life Research: Int J Qual Life Aspects Treat Care Rehabilitation. 2004;13(1):257–62. 10.1023/B:QURE.0000015295.74812.06 15058806

[24] Broadbent E, Petrie KJ, Main J, Weinman J. The brief illness perception questionnaire. J Psychosom Res. 2006;60(6):631–7. 16731240 10.1016/j.jpsychores.2005.10.020

[25] Zhang N, Fielding R, Soong I, Chan KK, Lee C, Ng A, et al. Psychometric assessment of the Chinese version of the brief illness perception questionnaire in breast cancer survivors. PLoS ONE. 2017;12(3):e0174093. 28319160 10.1371/journal.pone.0174093 PMC5358881

[26] Li Q, Lin Y, Hu C, Xu Y, Zhou H, Yang L, et al. The Chinese version of hospital anxiety and depression scale: psychometric properties in Chinese cancer patients and their family caregivers. Eur J Oncol Nursing: Official J Eur Oncol Nurs Soc. 2016;25:16–23. 10.1016/j.ejon.2016.09.004 27865248

[27] Zigmond AS, Snaith RP. The hospital anxiety and depression scale. Acta Psychiatrica Scandinavica. 1983;67(6):361–70. 6880820 10.1111/j.1600-0447.1983.tb09716.x

[28] Chen Z, He G, Zhao Y, Han C, Xu L, Jian H, et al. Symptom burden and emotional distress in advanced lung cancer: the moderating effects of physicians’ communication skills and patients’ disease understanding. Supportive Care Cancer: Official J Multinational Association Supportive Care Cancer. 2022;30(11):9497–505. 10.1007/s00520-022-07323-9 35971009

[29] Wang GL, Hsu SH, Feng AC, et al. The HADS and the DT for screening psychosocial distress of cancer patients in Taiwan. Psycho-oncology. 2011;20(6):639–46. 10.1002/pon.1952 21626611

[30] Wang H, Kindig DA, Mullahy J. Variation in Chinese population health related quality of life: results from a EuroQol study in beijing, China. Qual Life Research: Int J Qual Life Aspects Treat Care Rehabilitation. 2005;14(1):119–32. 10.1007/s11136-004-0612-6 15789946

[31] Luo N, Liu G, Li M, Guan H, Jin X, Rand-Hendriksen K. Estimating an EQ-5D-5L value set for China. Value Health: J Int Soc Pharmacoeconomics Outcomes Res. 2017;20(4):662–9. 10.1016/j.jval.2016.11.016 28408009

[32] Aiken LS, West SG. Multiple regression: Testing and interpreting interactions. Thousand Oaks, CA, US: Sage Publications, Inc; 1991. xi, 212-xi, p.

[33] Ünal Ö, Akyol Y, Tander B, Ulus Y, Terzi Y, Kuru Ö. The relationship of illness perceptions with demographic features, pain severity, functional capacity, disability, depression, and quality of life in patients with chronic low back pain. Turk J Phys Med Rehabil. 2019;65(4):301–8. 31893266 10.5606/tftrd.2019.3248 PMC6935732

[34] de Rooij BH, Thong MSY, van Roij J, Bonhof CS, Husson O, Ezendam NPM. Optimistic, realistic, and pessimistic illness perceptions; quality of life; and survival among 2457 cancer survivors: the population-based PROFILES registry. Cancer. 2018;124(17):3609–17. 30192384 10.1002/cncr.31634

[35] Asai K, Hatamochi C, Minamimura F. Association between illness perception and care-seeking intention in patients with chronic heart failure. Clin Nurs Res. 2023;32(3):669–76. 35934946 10.1177/10547738221114710

[36] Stanton AL, Danoff-Burg S, Cameron CL, Bishop M, Collins CA, Kirk SB, et al. Emotionally expressive coping predicts psychological and physical adjustment to breast cancer. J Consult Clin Psychol. 2000;68(5):875–82. 11068973

[37] Weldam SWM, Schuurmans MJ, Zanen P, Heijmans MJWM, Sachs APE, Lammers J-WJ. The effectiveness of a nurse-led illness perception intervention in COPD patients: a cluster randomised trial in primary care. ERJ Open Res. 2017;3(4). 10.1183/23120541.00115-2016 PMC5722077 29250529

[38] Dalili Z, Bayazi MH. The effectiveness of mindfulness-based cognitive therapy on the illness perception and psychological symptoms in patients with rheumatoid arthritis. Complement Ther Clin Pract. 2019;34:139–44. 30712718 10.1016/j.ctcp.2018.11.012

